# Medical Telemonitoring for the Management of Hypertension in Older Patients in Japan

**DOI:** 10.3390/ijerph20032227

**Published:** 2023-01-26

**Authors:** Takeshi Fujiwara, James P. Sheppard, Satoshi Hoshide, Kazuomi Kario, Richard J. McManus

**Affiliations:** 1 Nuffield Department of Primary Care Health Sciences, University of Oxford, Oxford OX2 6GG, UK; 2 Division of Cardiovascular Medicine, Department of Medicine, Jichi Medical University School of Medicine, Shimotsuke 329-0498, Japan

**Keywords:** aging, blood pressure, hypertension, medical telemonitoring, Japan

## Abstract

Hypertension is the most frequent modifiable risk factor associated with cardiovascular disease (CVD) morbidity and mortality. Even in older people, strict blood pressure (BP) control has been recommended to reduce CVD event risks. However, caution should be exercised since older hypertensive patients have increased physical vulnerability due to frailty and multimorbidity, and older patients eligible for clinical trials may not represent the general population. Medical telemonitoring systems, which enable us to monitor a patient’s medical condition remotely through digital communication, have become much more prevalent since the coronavirus pandemic. Among various physiological parameters, BP monitoring is well-suited to the use of such systems, which enable healthcare providers to deliver accurate and safe BP management, even in the presence of frailty and/or living in geographically remote areas. Furthermore, medical telemonitoring systems could help reduce nonadherence to antihypertensive medications and clinical inertia, and also enable multi-professional team-based management of hypertension. However, the implementation of medical telemonitoring systems in clinical practice is not easy, and substantial barriers, including the development of user-friendly devices, integration with existing clinical systems, data security, and cost of implementation and maintenance, need to be overcome. In this review, we focus on the potential of medical telemonitoring for the management of hypertension in older people in Japan.

## 1. Introduction

Aging is an inevitable part of life, is irreversible and is becoming a worldwide problem as people live longer [1]. Age-related changes in physical function make it more difficult to maintain healthy lives, limit physical activity, and increase the risk of disability [2]. Those biological degenerative changes and dysfunctions increase the clinical vulnerability of the older population [3]. Therefore, multi-dimensional health management interventions are needed to support healthy aging in older people.

Older populations are generally more likely to have multimorbidity and experience polypharmacy. Among the targets for improving the health of older people, hypertension is the most frequent and modifiable risk factor [4]. In Japan, according to the National Health and Nutrition Survey 2016, the prevalence of hypertension rises from 41–60% for people aged 40–74 years and to 74–77% for those aged ≥75, and is predicted to increase in the future as the population undergoes further aging [5,6,7]. Relative treatment benefits for hypertension are broadly consistent even as people age [8,9], but such treatment is often difficult due to problems such as frailty, nonadherence, cognitive impairment, as well as the risk of falls and postural hypotension which also increase with age. Judging carefully for each patient whether antihypertensive medications would be beneficial or harmful is crucial for the treatment of hypertension in older people.

In recent years, healthcare technology has remarkably progressed and our pursuit of health has changed dramatically, particularly in light of the coronavirus pandemic. Medical telemonitoring, defined as a continuous or non-continuous monitoring process that allows a healthcare professional to remotely manage a patient’s medical follow-up through digital communication [10], has become indispensable for healthcare. Among various physiological indicators that can be digitally captured, BP monitoring and subsequent management of hypertension are particularly suited to the use of digital health solutions [11,12,13,14]. These enable accurate and automatic exchange of BP values between healthcare professionals and their patients, which could be key to solving the current problems of nonadherence to antihypertensive medications and clinical inertia, and helpful for judging whether the antihypertensive medication use is appropriate or inappropriate. Older people would theoretically benefit significantly from BP telemonitoring; however, little evidence regarding the effectiveness of medical telemonitoring exists in this group [15], and implementation of medical telemonitoring systems in clinical practice has been slow.

In this review, we will examine the current evidence for BP management in older people and consider future developments in medical telemonitoring. In addition, we will discuss the prospects for BP management for older people in Japan using digital health solutions.

## 2. Methods

This narrative review aims to address and discuss the current evidence on medical telemonitoring systems for the management of hypertension, especially in older people in Japan. Key publications in the field were identified using PubMed and Google Scholar searches conducted in October 2022 using the search terms “medical telemonitoring” or “blood pressure telemonitoring” or “digital health” or “digital intervention” and “older”, or “elderly” and “hypertension”. The search was restricted to papers published in English and Japanese without time limit. Reference lists of articles identified in the search were also reviewed to identify additional relevant publications. For information on global demographic variables, including Japan, we searched them using Google (including Japanese).

## 3. Current Evidence for Hypertension Management in Older People

The Hypertension in the Very Elderly Trial (HYVET) was a landmark randomized clinical trial (RCT) of hypertension treatment focused specifically on those aged ≥80 years. The trial unequivocally showed the benefits of lowering BP in reducing the risks of stroke, all-cause mortality, and heart failure (HF) in older patients when treated to a target of ≤150/80 mmHg [16]. In recent years, it has been shown that even stricter BP control can be beneficial in older patients. In the subgroup of patients aged ≥75 years who enrolled in the Systolic Blood Pressure Intervention Trial (SPRINT), the intensive treatment group (systolic BP [SBP] target of <120 mmHg, automated unattended BP measurement) showed significantly lower rates of cardiovascular disease (CVD) outcomes (hazard ratio [HR] 0.66, 95% confidence interval [CI] 0.51 to 0.85) and all-cause mortality (HR 0.67, 95% CI 0.49 to 0.91) compared with the standard treatment group (SBP target of <140 mmHg) [8]. Achieved BPs as measured in the clinic were, however, significantly above these targets. The Strategy of Blood Pressure Intervention in the Elderly Hypertensive Patients (STEP) trial also showed the clinical benefits of strict SBP control, at least in those aged 60-80 years [9]. The intensive treatment group (SBP target of 110 to <130 mmHg) showed a significantly lower incidence of the total CVD event than the standard treatment group (SBP target of 130 to <150 mmHg) (HR 0.74, 95% CI 0.60 to 0.92). Among the components of total CVD events, the greatest risk reduction was observed in the incident acute decompensated HF (HR 0.27, 95% CI 0.08 to 0.98). In a sub-analysis of the nationwide practice-based prospective Japan Morning Surge-Home Blood Pressure (J-HOP) study on mostly medicated 349 hypertensive patients aged ≥80 years, the morning home BP showed a positive linear association with CVD events, especially with stroke [17]. The most recent meta-analysis of the Blood Pressure Lowering Treatment Trialists’ Collaboration (BPLTTC), which included 51 RCTs with 358,707 participants, also showed that pharmacological BP-lowering treatment reduced the risk of major CVD events, including stroke, ischemic heart disease, and HF, regardless of age, down to less than 120/70 mmHg [18].

On the other hand, potential risks of hypertension treatment in older people should also be considered. The use of antihypertensive medications in older patients may be associated with adverse drug reactions and drug–drug interactions [19]. Intensive BP therapy in older patients may be harmful [20], increasing the risk of falls [21], syncope [22], fractures [23], acute kidney injury, and electrolyte abnormalities [24,25,26]. In addition, caution is needed when extrapolating the results of clinical trials to older people in clinical practice. It should be noted that SPRINT excluded patients with diabetes and stroke, which are common among older people, and that automated office BPs were used for the assessment of the risks between CVD outcomes and BP level [8]. Automated office BP is a fully automated unattended BP measurement technique, which has been designed to record multiple BP readings in the office, with the patient resting alone in a quiet room (no presence of medical staff), and reduce the white-coat effect associated with manual office BP measurement [27]. Automated office BP values were lower than physician’s office BP (SBP, −10.48 mmHg) and non-physician’s office BP (SBP, −6.89 mmHg) [28], which meant that it is difficult to determine which office BP levels to target in clinical practice. Furthermore, the long-term effect of intensive BP lowering has now been called into question. In a recently reported long-term follow-up of the SPRINT (4.5 years of post-trial observation), the intensive treatment group showed an increase in SBP from 132.8 mmHg at five years following randomization to 140.4 mmHg at 10 years. Moreover, the benefits of intensive BP lowering were no longer evident for CVD outcomes (HR 1.02, 95% CI 0.84 to 1.24) and all-cause mortality (HR 1.08, 95% CI 0.94 to 1.23) compared to the standard treatment group [29].

It is also important to note that most patients aged ≥80 years who participated in RCTs would differ from the general population aged ≥80 years. In particular, those with frailty, multiple cardiovascular medications, and multimorbidity are less likely to be eligible [30]. For example, SPRINT excluded by design around 2:3 people over 80. Among older Japanese patients with isolated systolic hypertension, a U-shaped relationship was observed between the achieved SBP levels and the risks of CVD events and all-cause mortality, that is, the risks of them were minimized in achieved SBP levels of 130 to <145 mmHg, but increased in achieved SBP levels of <130 mmHg and ≥145 mmHg [31]. The results of this study imply that excessive reduction of SBP in older people might increase the risk of CVD events and all-cause mortality. Due to the complex and heterogeneous pathological nature of hypertension in older people, further studies are warranted to confirm the efficacy and safety of pharmacological BP-lowering treatment for CVD risk reduction in older people.

## 4. State-of-the-Art Evidence of BP Telemonitoring for the Management of Hypertension

Digital technologies have potential for transforming healthcare across multiple dimensions, including quality, access, patient experience, and cost effectiveness. Indeed, recent evidence demonstrates the feasibility, acceptability, and success of medical telemonitoring in changing the behavior of people with chronic conditions [32,33,34,35,36,37,38]. Among the management of chronic conditions, hypertension is a good target for medical telemonitoring.

For the last 20 years, the effectiveness of BP telemonitoring systems have been evaluated [39,40,41]. After the coronavirus pandemic, a significant demand for digital healthcare and medical telemonitoring has grown rapidly around the world [42]. In response to those trends, the management of hypertension using digital healthcare technology seems likely to continue in this direction of travel [43].

In the UK population of 622 hypertensive patients with treated but poorly controlled (>140/90 mmHg), the Home and Online Management and Evaluation of Blood Pressure (HOME BP) trial showed the potential of digital interventions, combined with self-monitoring, for the management of hypertension [44]. Compared with the usual care group (n = 317, mean age 66.7 ± 10.2 years; including routine hypertension care, with appointments and antihypertensive medication changes made at the discretion of the general practitioner), the digital intervention group (n = 305, mean age 65.2 ± 10.3 years; including BP self-monitoring, self-titration of antihypertensive medications based on self-monitored BP levels, lifestyle advice, and behavioral support for patients and healthcare professionals) showed a significant reduction of office SBP after 12 months (−3.4 mmHg, 95% CI −6.1 to −0.8 mmHg). In a Japanese population of untreated patients with essential hypertension, the HERB Digital Hypertension 1 (HERB-DH1) pivotal trial also highlighted the potential effects of a digital intervention for non-pharmacological lifestyle modification to reduce BP levels [45]. Untreated patients with hypertension (office SBP 140 to <180 mmHg, and 24-h ambulatory SBP ≥130 mmHg) were randomly assigned to the digital therapeutics group (n = 199, mean age 52.4 ± 8.1 years; the HERB system with standard lifestyle modification) or the control group (n = 191, mean age 52.0 ± 7.6 years; standard lifestyle modification alone). In the digital therapeutics group, patients input their demographic profiles and home BP values via the HERB application downloaded to their smartphone. Based on these data, the HERB system generated a personalized program of lifestyle modifications designed to reduce BP. At 12 weeks, compared with the control group, the digital therapeutics group showed significant reductions from baseline in 24-h ambulatory, morning home, and office SBP; between group differences −2.4 mmHg (95% CI −4.5 to −0.3 mmHg), −4.3 (95% CI −6.7 to −1.9 mmHg), and −3.6 mmHg (95% CI −6.2 to −1.0 mmHg), respectively. Even after addition of antihypertensive therapy being permitted from 12 weeks, the difference at 24 weeks remained significant. In addition, these trials also showed that digital interventions can be cost-effective compared to conventional treatment for reducing BP levels [46].

In an individual patient data meta-analysis of 15 studies including a total of 7138 individuals, self-monitoring of BP with co-intervention (including systematic medication titration by doctors, pharmacists, or patients; education; or lifestyle counselling, using telemonitoring systems) showed significant BP reduction compared to the usual care at 12-months follow-up [47]. In the subgroup analyses by age and comorbidities, the effects of self-monitoring with co-intervention were similar [48].

## 5. Cardiovascular Disease and Hypertension: Current Status in Japan

Japan has one of the longest life expectancies in the world for both men and women (men 82 years, women 88 years) [49]. However, this is whole life and not healthy life expectancy which is critically important for both personal quality of life (QOL) and public health. In the annual report of the World Health Statistics 2022, healthy life expectancies in Japan were considerably lower, at 72.6 years in men and 75.5 years in women [50], meaning that people in Japan have to live with some kind of disability for approximately the last 10 years of their lives. To maintain or extend the sustainability of health under aging populations in Japan, it is important to understand the underlying causes of this deterioration in later life.

According to the annual report 2019 from the Ministry of Health, Labor, and Welfare in Japan, stroke and dementia are the leading causative diseases requiring long-term care, including being bedridden, with these two diseases accounting for about 50% of all cases [51]. Hypertension is a significant risk factor for both the onset of stroke and for the development of dementia [52,53,54,55,56,57]. The Evidence for Cardiovascular Prevention From Observational Cohorts in Japan (EPOCH-JAPAN), a meta-analysis of 10 cohort studies including 67,309 Japanese individuals, showed that increased BP has been shown to be positively associated with increased risk of long-term CVD, even in very old people (age 75 to 89 years) [58]. Patients with CVD experience numerous physical symptoms including fatigue, dyspnea, or chest pain which affect their physical, emotional, and social well-being with significant impairment in QOL [59]. Among CVD, HF in older people is a major public health problem significantly reducing activities of daily living and closely linked to hypertension [60,61,62,63,64].

It is evident that hypertension therefore increases CVD risk and reduces QOL for older people; however, the control rate of hypertension in Japan for the elderly (age 70 to 79 years) remained only 44.2% among men and 43.4% among women in 2016 [65]. Various factors, such as high salt intake/sodium sensitivity, autonomic dysfunction, obesity, multimorbidity, frailty, nonadherence, and sleep disorders might affect the insufficient control rate of hypertension, but lower rates of awareness and insufficient treatment of hypertension might also contribute [65]. The Japanese population, including older people, need to change their perception of hypertension, and healthcare providers also need to recognize and fight the pervasive problem of clinical inertia [66]. Since BP management in older people is crucial for reducing the public health burden in Japan, measures to reduce the number of poorly controlled hypertensive patients are urgently needed.

## 6. Implementation of Medical Telemonitoring for Older People with Hypertension in Japan

**Figure 1:**
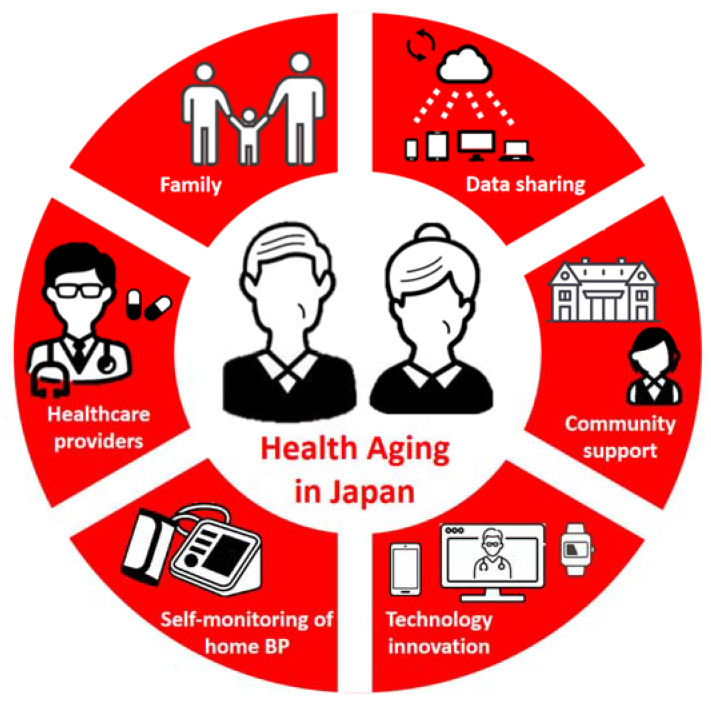
Patient-centered management of hypertension in older people using digital healthcare solutions in Japan.

The traditional approach, in which only physicians make decisions for the patients’ BP control, neglects modern scientific and technological advances, and this approach does not address either patients’ nonadherence to antihypertensive medications or the clinical inertia of healthcare providers. Modern healthcare systems require a shift toward patient-centered care, where the patient’s goals and wishes are respected in determining the treatment, with increasing awareness of evidence-based self-management (Figure 1) [48,67,68].

The table (Table 1) summarizes the advantages and disadvantages of using BP telemonitoring systems for the management of hypertension in older people [13]. To date, BP telemonitoring has mostly been used in clinical trials and has not yet been widely used in clinical practice. This section discusses the benefits of medical telemonitoring systems for the management of hypertension in older people and the current barriers to its widespread use in Japan.

### 6.1. Blood Pressure Measurement

Accurate measurement of BP is particularly needed in older people, since any over or underestimation of BP could expose them to either an increased risk of CVD events or of adverse events, such as falls and postural hypotension [69]. Using a standardized recording and transmission system allows physicians to assess all available BP values and automatically calculate mean BP level, which could help to determine whether antihypertensive medications should be up-titrated or withdrawn. BP management using telemonitoring systems can provide patients with a feeling of security and invoke greater interest in their BP levels [70,71]. Capturing out-of-office BP using a telemonitoring system can help to identify white-coat and masked hypertension, which are common in older people [72,73]. It can also help to detect seasonal variations in BP that can be associated with increased CVD risks of older people in the winter [74,75,76].

On the other hand, many challenges remain to be overcome to disseminate medical telemonitoring systems in clinical practice. The development of simple devices that can measure BP accurately in all age groups is desirable. To use such devices, continuous training and education about how the devices work are necessary. It is also necessary to establish a robust social system for data security and privacy protection of individuals.

Some patients may feel anxiety with remote management, and this can lead to frequent repeating of BP measurements. It may also give the impression of constant monitoring by physicians, which can feel excessive to some patients. For physicians, unless patients use devices that can automatically transfer measured BP to the clinical record, they would have to manually enter the BP values measured by patients, resulting in increased workload. In addition, physicians often need to log on to standalone websites as an additional step in order to check the patients’ data whereas integrated systems would avoid this. A qualitative study implied that traditional paper-diary methods may suit existing clinical practices compared to parallel BP management using telemonitoring systems, in the absence of appropriate integration [70]. Thus, increasing the generalizability of medical telemonitoring systems is likely to depend on their interoperability with electronic health records, especially for generalists who could otherwise conceivably require several different telemonitoring systems for the range of clinical problems they see.

### 6.2. Frailty

Frailty is a common condition among older people and becomes more prevalent with age. The progression of frailty increases the vulnerability of all bodily functions, including generalized muscle weakness, low vision, and hearing problems. These can make it difficult for older people to keep an accurate record of their home BP levels and lead to both mis-reporting and complete lack of reporting (up to 50% may not inform their primary care physician of their self-monitoring) [77,78,79]. Older populations may also have lower levels of technology literacy [70,80] and/or require the support of carers. Notwithstanding the issues of standalone systems, we have previously demonstrated the high feasibility of recording home BP using BP telemonitoring by older hypertensive patients (mean age 76.4 ± 7.8 years) in real-world clinical settings as a solution to these issues [81]. Digital solutions can also facilitate team-based approaches in which not only physicians but also nurses/public health nurses, pharmacists, care workers, dieticians, and other multidisciplinary professionals utilize their expertise and share their information [82,83].

### 6.3. Nonadherence

Nonadherence to antihypertensive medication is common, especially in patients with uncontrolled BP or treatment resistance [84,85]. Nonadherence is also associated with higher risk of future CVD events [86]. In addition, nonadherence is a significant cost burden on healthcare systems [87]. Over half of older people are estimated to suffer from multimorbidity [88], which usually requires complex medical treatment and can lead to subsequent poor adherence [89]. Adherence to antihypertensive medication is essential to achieve therapeutic benefits and management of hypertension [90,91].

Digital healthcare approaches can be useful for detecting nonadherence and for improving adherence. Mobile phone text messaging and applications can improve medical adherence in chronic disease [92,93]. A meta-analysis which evaluated the effectiveness of mobile application-based interventions on medication adherence and BP levels in patients with CVD also showed that application-based interventions could improve SBP and diastolic BP levels to controls [94]. In recent years, the use of digital pill systems has been reported to be useful in directly measuring adherence and may become more widespread in the future [95,96].

These innovative and device-based strategies are useful tools for increasing drug adherence but should be combined with reduced complexity of antihypertensive regimes for the management of hypertension in older people [97]. Reducing or avoiding medications that can increase BP, such as non-steroidal anti-inflammatory drugs, corticosteroids, some anti-rheumatic drugs, and sympathomimetic drugs, are other interventions that can be taken when possible [98]. Educational approaches for patients on the importance of taking antihypertensive medication regularly and on changing misconceptions about hypertension and antihypertensive medications, such as stress-related conditions, including headache, palpitations, and dizziness, and fear of addiction or dependence on drugs, are also important [99]. Healthcare professionals must have many opportunities to discuss therapeutics with patients for a greater understanding.

### 6.4. Clinical Inertia

Clinical inertia, defined as a failure of healthcare providers to initiate or intensify therapy when indicated [100], is one of the most challenging problems in the current management of hypertension. Clinical inertia is due to at least three problems: overestimation of care provided; use of “soft” reasons to avoid intensification of therapy; and lack of education, training, and practice organization aimed at achieving therapeutic goals [100]. In a recent general practitioner-based cohort study using electronic health record data in the Netherlands, clinical inertia was reported to be the cause in 87% of cases of uncontrolled BP [101]. Older age, readings closer to target BP, and concurrent diabetes were significantly related to clinical inertia [101]. Most general practitioners in this study considered near-target BP values acceptable in most cases for everyday primary care. BP telemonitoring systems can help reduce such inertia by automatically providing comprehensive estimates of mean BP level [102].

In Japan, significant differences in perceptions of hypertension management between physicians and patients have been reported in the web-based survey: approximately 80% of physicians reported that they had fully or sufficiently provided education to patients about reasons for hypertension treatment and its associated risks, target BP levels, and lifestyle modifications; however, only 40–50% of patients considered those topics having been fully or sufficiently discussed [103]. Medical telemonitoring systems can provide both patient education and improved communication with physicians [70], and their active use could help bridge these perception gaps between physicians and patients.

### 6.5. Monitoring of Adverse Events

Treatment benefits for hypertension are broadly consistent across age groups [18,104]. However, in the recent meta-analysis of 58 RCTs including a total of 280,638 individuals, antihypertensive treatments were associated with an increased risk of acute kidney injury, hyperkaliemia, hypotension, and syncope [26]. As physiological reserve declines in older people, healthcare professionals should carefully monitor adverse effects and consider withdrawing inappropriate antihypertensive medications in those at high risk of harm [105].

There is still a lack of definitive evidence for monitoring adverse events of antihypertensive medications using telemonitoring systems; however, those systems would have many potential uses in routine clinical practice. Telemonitoring systems could enable monitoring of excessive BP decreases or postural hypotension and reduce the risk of syncope and falls. An innovative telemonitoring device that allows both actigraphy function and BP measurement could assess the frequency of falls in the home and their relationship to BP levels, which could aid the appropriate withdrawal of antihypertensive medications [106]. Short-term BP assessment using telemonitoring systems after the withdrawal of antihypertensive medication may also help to determine whether the decision was appropriate. Indeed, this was used effectively in the recent Optimising Treatment for Mild Systolic Hypertension in the Elderly (OPTIMISE) antihypertensive deprescribing trial [107], where participants were given the opportunity to monitor their own BP following antihypertensive drug withdrawal, enabling those experiencing large increases in BP to flag this to their general practitioner and in some cases, have their medication reinstated.

### 6.6. Geographically Remote Rural Areas

Japan is comprised of nearly 7000 islands. In addition, approximately 60% of the area in Japan is identified as underpopulated or remote, where 8.6% of the total population (approximately 12 million people) live. In such areas, the proportion of older population aged ≥65 years is high at 36.7% in 2017 [108]. In addition, the proportion of older people is expected to continue to increase, including those living alone or living with an older spouse only.

There are significant cultural, social, and economic differences between rural and urban areas, and frequent visits to health facilities are often difficult to achieve. Indeed, awareness, treatment, and control rates of hypertension were lower in rural areas compared with urban ones [109]. There is an urgent need for healthcare promotion for hypertension in such areas where medical resources are scarce.

BP telemonitoring systems have the potential to make a significant contribution to controlling BP in geographically isolated settings [110], since they can allow healthcare professionals to access a patient’s BP levels remotely [71]. In addition, such systems could reduce the frequency of unnecessary office visits. In older people living alone, a medical telemonitoring system could be used to manage their health, which could also be used as a confirmation of safety status from remote areas. In such geographically remote areas, mobile technologies, such as mobile phones and tablet computer applications, could be particularly useful for self-management of hypertension since new equipment for internet facilities and its regular maintenance can be costly. Medical telemonitoring systems have the potential to enable high quality healthcare to be easily accessible at low cost, even in areas previously referred to as medically underserved [111,112].

### 6.7. Patients Living in Nursing Care Facilities

Approximately 0.76% of the total population in Japan (almost a million people) live in nursing care facilities as of 2022 and this is expected to continue to increase in the future [113]. A previous study demonstrated that over 60% of patients living in nursing homes had diagnosed hypertension and over 90% of patients took antihypertensive treatments [114]. Since multimorbidity increases the risks of adverse events, frailty, cognitive impairment, and polypharmacy are major concerns among patients living in nursing care facilities [115]; intensified attention would be needed in management of hypertension.

Observational studies in older people living in nursing care facilities have shown that intensive antihypertensive treatments may lead to paradoxical increase in CVD hospitalization and mortality [116,117,118]. Therefore, it is also necessary to positively consider withdrawal of antihypertensive medications for very old patients for whom competing risks may also be important. Particularly those near the end-of-life stage who are living in nursing care facilities, based on comprehensive patient assessments. The use of medical telemonitoring systems could be useful for identifying those with excessive BP decreases and for medication titration. Using wearable technology, biometric information, including BP levels, could be obtained non-invasively and continuously, even in those with reduced mobility or even bedridden patients. Further interventional studies are warranted to explore benefits or harms of antihypertensive medications in patients living in nursing care facilities, and we believe that medical telemonitoring systems would be beneficial in the management of hypertension in this growing population.

## 7. Conclusions

This review has summarized the current evidence on medical telemonitoring systems for the management of hypertension in older people. Recent evidence on the management of hypertension in older people suggests that strict BP control would be beneficial for reducing CVD risks, and in this regard, BP telemonitoring systems could be useful to provide more accurate and safer BP management in older people. On the other hand, physicians must monitor adverse events carefully and consider the benefits or harms of antihypertensive medications in older people with frailty and multimorbidity. Medical telemonitoring systems have the potential to be a solution to the current problems of nonadherence and clinical inertia, and to help to judge whether the antihypertensive medication use is appropriate. However, there are many barriers that need to be resolved for wide use in clinical practice. Hardware improvements, such as development of user-friendly devices targeted at older people, and social infrastructure development, are essential, as is the establishment of multidisciplinary professional support systems utilizing digital healthcare solutions to support patient management.

Healthcare innovation is expected to further accelerate in the management of hypertension, as hypertension application-based treatment (CureApp HT, CureApp Inc. Tokyo, Japan) has been covered by insurance for the first time in September 2022 in Japan [119]. Healthcare professionals should carefully and fully discuss how these modern devices can be implemented into the existing healthcare delivery system. Further clinical trials are needed to clarify long-term CVD risk management and the issues to be implemented in the clinical practice of medical telemonitoring systems. These innovations and accumulation of evidence could lead to healthy aging in Japan (Figure 1).

## Figures and Tables

**Table 1:** Advantages and disadvantages of blood pressure telemonitoring system for the management of hypertension in older patients.

	Advantages	Disadvantages
**For physicians**		
	Assessment of whole blood pressure (BP) data sets	Need to provide simple and user-friendly devices
	Accurate/reliable BP data	Need maintenance of systems
	Easy to assess the mean BP data calculated automatically	May be time-consuming to input patients’ BP data
	BP levels can be determined quickly after changes in antihypertensive medication	Need to log onto websites for checking data
	Provision of personalized self-care recommendations	May require help of others to make the system work
	Saving time for consultation	Need to ensure data security and patients’ privacy
	Identification of white-coat hypertension and masked hypertension	Need to consider legal and ethical issues
	Assessment of seasonal BP variation	Need to interoperate with existing systems
	Medication adherence can be confirmed	
	Easy to share medical information and consult with specialists	
**For patients**		
	Good BP control	Additional costs to use data transmission system
	Increased motivation for BP measurements	Need to build an internet environment
	BP measured easily without the need to record BP levels in a logbook	Need training to use devices correctly
	Cost effective in reducing cardiovascular disease	Need to consider digital literacy
	Reduced frequency of office visits	May cause anxiety and too frequent monitoring
	Reduced risks of adverse events by antihypertensive medications	May give a sense of insecurity that they are constantly being monitored
	Rural locations or natural disaster-affected areas not a barrier	
	Family or physicians can confirm the safe status of patients	
	Easy to communicate with his/her healthcare professionals	

## Data Availability

Not applicable.
